# A Novel Xenograft Model in Zebrafish for High-Resolution Investigating Dynamics of Neovascularization in Tumors

**DOI:** 10.1371/journal.pone.0021768

**Published:** 2011-07-13

**Authors:** Chengjian Zhao, Xiaofei Wang, Yuwei Zhao, Zhimian Li, Shuo Lin, Yuquan Wei, Hanshuo Yang

**Affiliations:** 1 State Key Laboratory of Biotherapy and Cancer Center, West China Hospital, West China Medical School, Sichuan University, Chengdu, Sichuan, People's Republic of China; 2 Department of Molecular Cell and Developmental Biology, University of California Los Angeles, Los Angeles, California, United States of America; Cedars-Sinai Medical Center, United States of America

## Abstract

Tumor neovascularization is a highly complex process including multiple steps. Understanding this process, especially the initial stage, has been limited by the difficulties of real-time visualizing the neovascularization embedded in tumor tissues in living animal models. In the present study, we have established a xenograft model in zebrafish by implanting mammalian tumor cells into the perivitelline space of 48 hours old Tg(Flk1:EGFP) transgenic zebrafish embryos. With this model, we dynamically visualized the process of tumor neovascularization, with unprecedented high-resolution, including new sprouts from the host vessels and the origination from VEGFR2^+^ individual endothelial cells. Moreover, we quantified their contributions during the formation of vascular network in tumor. Real-time observations revealed that angiogenic sprouts in tumors preferred to connect each other to form endothelial loops, and more and more endothelial loops accumulated into the irregular and chaotic vascular network. The over-expression of VEGF165 in tumor cells significantly affected the vascularization in xenografts, not only the number and size of neo-vessels but the abnormalities of tumor vascular architecture. The specific inhibitor of VEGFR2, SU5416, significantly inhibited the vascularization and the growth of melanoma xenografts, but had little affects to normal vessels in zebrafish. Thus, this zebrafish/tumor xenograft model not only provides a unique window to investigate the earliest events of tumoral neoangiogenesis, but is sensitive to be used as an experimental platform to rapidly and visually evaluate functions of angiogenic-related genes. Finally, it also offers an efficient and cost-effective means for the rapid evaluation of anti-angiogenic chemicals.

## Introduction

Tumorgenic neovascularization is established as essential for sustaining tumor progression and metastatic spread, in response to interactions between tumor cells and endothelial cells, growth factors, and extracellular matrix components [1]–[5]. Without angiogenesis, most tumors will not progress to a clinically relevant size nor will they metastasize to distant organs through the blood stream. After the triggering of ‘angiogenic switch’ at the early stage of tumor growth, tumors vascular network will be established gradually. Vasculatures in tumors often are architecturally different from their normal counterparts — they are haphazardly constructed, irregularly shaped and tortuous [6]–[8]. As a result, blood flow is irregularly in tumor vessels, moving much slowly and sometimes even oscillating [9]. The structural and functional abnormalities in tumor vessels produce the unique tumor microenvironment — hypoxic and lacking nutrients, owning to poor blood perfusion, high interstitial fluid pressure, acidosis, fast growth and metabolic rates of malignant tissues [10], and finally promote tumor cells to invade adjacent healthy tissue and leading to metastasis—the major cause for failure in cancer treatment [11], [12]. The process of tumorgenic neovascularization is so complex that it has not been fully understood, although various signals that trigger this switch have been discovered. It is necessary to develop novel high-resolution experimental systems to further understand the abnormalities and underlying mechanisms of tumor vasculatures, and for the development of antiangiogenic chemicals.

An ideal experimental system for investigation of neovascularization in tumors, at least in our opinion, should posses these characteristics: the high-resolution on single-cell level, appropriate for real-time observation and quantitative analysis, can display the critical process of the transition from avascular to vascular stage, can be used for anti-angiogenesis drug discovery, easy to establish and manipulate in numerous animals, low costs for research. Some experimental models have been established in rodents and chick embryo to investigate the angiogenic biology and for the screening of proangiogenic and antiangiogenic compounds. The technological developments of skinfold chamber and intravital microscopy have deepened the understanding to tumor-induced angiogenesis at the early stage of tumor progression [3]–[5]. However, each model or technology has its advantages and disadvantages [13], [14], none of them simultaneously fulfill all requirements mentioned above.

Recently, the zebrafish has been developed as a promising experimental model for cancer research because of strikingly similar molecular and histopathological features between fish and human [15]–[17]. This animal model has many advantages in cancer research field comparing to other vertebrate model systems, such as transparency, ease of experimentation, feasibility to *in vivo* manipulation, and so on. Several studies have reported the approaches to investigate tumor growth, metastasis and interactions between tumor cells and neighboring vessels [18]–[21]. After transplantation of human tumor cell lines into zebrafish embryos, the neovascularization induced by tumor xenografts was observed [18], [19]. However, the tumor xenografts are embedded in the zebrafish tissues, it's difficult to clearly and dynamically monitor tumor growth and neovascularization within tumor tissues. In the present study, we described a novel experimental procedure to establish the tumor xenograft model in Tg(Flk1:EGFP) transgenic zebrafish, in which individual green endothelial cells can be clearly distinguished from red tumor cells. The process of neovasularization in tumors can be dynamically visualized and quantitatively analyzed on single-cell level. Moreover, this model is sensitive enough to respond to the alternation of angiogenic gene in tumor cells and small molecular antiangiogenic chemicals.

## Materials and Methods

### Cell and Cell Culture

B16 mouse melanoma cells, CT26 mouse colon cancer cells and human embryonic kidney cells HEK293 were obtained from American Type Culture Collection (ATCC, Manassas, VA). All cells were cultured at 37°C in 5% CO_2_ in DMEM supplemented with 10% fetal bovine serum. The stable transfection was generated with pCMV-DsRed-express (Clontech, USA) by using Lipofectamine 2000 reagent (Invitrogen, USA), followed by G418 selection. Clones expressing red fluorescence were isolated for further selection. Selected red fluorescence-labeled cells were cultured at 37°C in 5% CO_2_ in DMEM supplemented with 10% fetal bovine serum and 200 µg/ml selective agent.

### Ethics Statement

All animal work have been approved by Sichuan Animal Care and Use Committee and conducted according to relevant guidelines. The Permit Number is SYXK (Chuan) 2008-119.

### Zebrafish Husbandry and Cell microinjection

Tg(flk1: EGFP) zebrafish were bred and maintained normally (temperature, 28°C; pH 7.2–7.4; 14 hr on and 10 hr off light cycle). Wild type or red fluorescence-labeled cells were harvested at a concentration of 1×10^7^ cells/ml. This mixture was loaded into a borosilicate glass needle pulled by a Flaming/Brown micropipette puller (Narishige, Japan, PN-30). 5∼10 nanoliters suspension containing about 50–100 cells were implanted into each zebrafish embryo through the perivitelline space in a single injection by using an electronically regulated air-pressure microinjector (Harvard Apparatus, NY, PL1-90). After injection, zebrafish were washed once with fish water and examined for the presence of fluorescent cells. For each implantation, about 50 fish were selected and transferred to 6-well plate containing 2 ml of fresh fish water and subsequently documented photographically. Fish water was changed daily, and larva that more than 5 days old were fed twice a day with grinded brine shrimp and maintained under normal fish husbandry conditions.

### Imaging

Living zebrafish embryos were anesthetized by 0.003% tricaine and embedded in a dorsal, ventral, or lateral orientation in 3% methylcellulose. Digital micrographs were taken with a Zeiss Imager.Z1 fluorescence microscope (Carl Zeiss Microimaging Inc., Germany) equipped with an AxioCam MRc5 digital CCD camera (Carl Zeiss Microimaging Inc., Germany). Whole animal images were taken with a Zeiss Stemi 2000-C stereomicroscope with the AxioCam MRc5 digital CCD camera (Carl Zeiss Microimaging Inc., Germany). All images were taken in the same focal plane in brightfield and intransmitted light passing through RFP or GFP filters. Excitation was 488 nm for GFP, 561 nm for DsRed. To clearly imaging all vessels within xenograft models, 0.5–2 µm step z-stacks (512×512 focal planes, 50–200 µm in depth) were acquired by using 10×(ZEISS, Plan-Neofluar) or 20×(ZEISS, Plan-Neofluar) objectives. Images capture, processing and adjustment were performed with ZEISS Axiovision rel.4.8 software.

### Pharmacological Treatment of Fish with SU5416

SU5416 (Sigma) was added directly into the fish water at a final concentration of 2 µM. DMSO (Sigma) was used as a vehicle control. Animals were maintained in 2 ml fish water that was changed daily.

### Quantitative analysis of neovascularization in tumor xenografts

Measurement was done on the zebrafish photos taken with a Zeiss Imager.Z1 fluorescence microscope (Carl Zeiss Microimaging Inc., Germany) equipped with an AxioCam MRc5 digital CCD camera (Carl Zeiss Microimaging Inc., Germany). The tumor size, vessel length and vessel diameter were quantified by Axiovision Rel 4.8 software (Carl Zeiss Microimaging Inc., Germany).

### MTT assay

The growth rate of B16 cells *in vitro* was determined by the 3-(4,5-dimethylthiazol-2-yl)-2,5-diphenyl tetrazolium bromide (MTT; Sigma, St. Louis, MO, USA) colorimetric assay. Briefly, about 3000 cells were cultured in a 96-well plate at a volume of 100 µL of medium/well and incubated over night. The cells were incubated in complete medium containing 0, 1, 2 µM SU5416, respectively. After 48 h of incubation, the medium was aspirated and 20 µL of 5 mg/mL MTT was added per well and incubated at 37°C for 4 h; then supernatant fluid was removed, and 150 µL of dimethyl sulfoxide (DMSO) was added per well. Spectrometric absorbance at 490 nm was measured using a microplate reader. The cell survival rate was assessed as percent cell viability in terms of non-treated control cells.

### Western blotting

B16 cells were planted in six-well plate (about 5×10^5^ per well) and transfected pcDNA3.1- VEGF165 or pcDNA3.1 vector with Lipofectamine 2000 (Invitrogen) as the standard protocol. Forty-eight hours after transfection, western blot analysis was performed [22] to test the expression of VEGF165 in B16 cells. Briefly, total lysate proteins of B16, B16-vector, B16-VEGF165 cells were separated on SDS- SDS-polyacrylamide gels and transferred to a PVDF membrane. After transfer, the membrane was blocked with 5% nonfat dry milk in PBS with 0.5% Tween 20 to block nonspecific protein binding. The protein of interest (VEGF or β-actin) were identified by incubating with monoclonal antibody in TBS with 0.1% Tween and 5% nonfat milk (blotting buffer) overnight at 4°C. β-actin (Santa Cruz) was detected as the internal control. After several washing, PVDF membrane was incubated with horseradish peroxidase–conjugated secondary antibody (1∶8,000) in blotting buffer at room temperature for 45 minutes. Then membranes were washed and protein antibody complexes were visualized by chemiluminescent detection using SuperSignal West Pico Chemilumenescent Substrate (Pierce).

### Histology Analysis

To evaluate the tumor growth on zebrafish embryos, the xenografted zebrafish was embedded in tissue freezing medium (OCT). Frozen zebrafish was sectioned to 8 µm thickness, and hematoxylin and eosin staining was performed as standard protocol.

### Statistical Analysis

Data was assayed by unpaired student's t test using SPSS18.0 statistical analysis software. A level of P<0.05 was regarded as statistically significant.

## Results

### The establishment of tumor xenograft models in zebrafish

**Figure 1:**
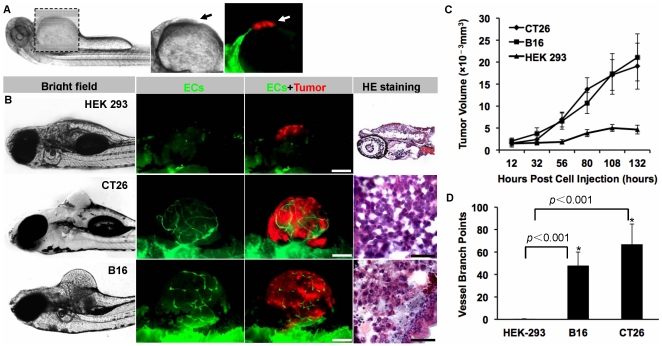
The establishment of mammalian tumor xenograft models in zebrafish. Red fluorescence-labeled malignant tumor cells (murine colon carcinoma CT26 and melanoma B16 cells) and non-tumorigenic HEK 293 cells were implanted into the abdominal perivitelline space (A, indicated by arrow) of 48 hpf Tg(flk1:EGFP) transgenic zebrafish embryos (50–100 cells/embryo). When tumors (B, red) reached 350–450 µm in diameter when filled with neo-vessels (B, green), the neoplasia were isolated and analyzed by H&E staining (B). The growth rate of xenografted tumors (C) and the number of tumor-induced neovessels (D) were analyzed quantitatively. * indicates the significant difference. Scale bar, 100 µm.

Red fluorescence-labeled malignant cells (murine colon carcinoma CT26 and melanoma B16 cells) were implanted into the perivitelline space of 48 hpf (hours post fertilization) Tg(flk1:EGFP) transgenic zebrafish embryos (50–100 cells/embryo). Non-tumorigenic HEK 293 cells were injected as the control (Fig. 1A,B). Five days after cells injection, solid tumors (350–450 µm in diameter) were established in 100% of CT26 and B16 cells implants (n = 50). The formed neoplasia with hemispheric morphology obviously protruded from the abdomen of the transgenic zebrafish (Fig. 1B). Dual fluorescent living images demonstrated a plenty of green neovessels existed in red tumor cells (Fig. 1B). On the contrary, non-malignant HEK 293 cells failed to form any obvious tumors on the fish abdomen and no endothelial cells penetration was observed as well (Fig. 1B). The HE staining of neoplasia tissues further confirmed the establishment of B16 and CT26 tumor in the zebrafish embryos (Fig. 1B). Meanwhile, the growth rate of the xenografted tumor and the number of tumor-induced neovessels were quantified, showing the significant difference between malignant cells (B16 and CT26) and non-malignant HEK 293 cells (P<0.001) (Fig. 1C,D).

### The quantitative analysis of different neovascularization modes

**Figure 2:**
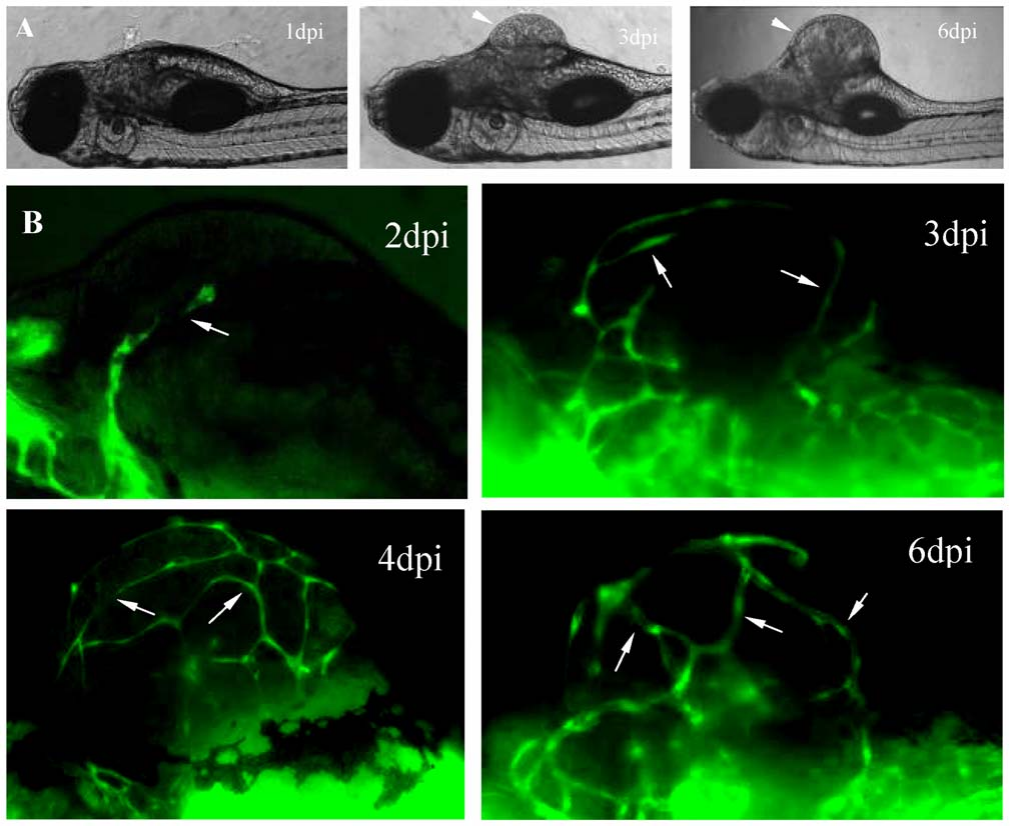
Dynamical observations of aniogenesis in B16 xenografts. The established microtumors (A, 3dpi and 6dpi panels) in zebrafish larvae are indicated by arrowheads. Neo-vessels are indicated by arrows (B).

**Figure 3:**
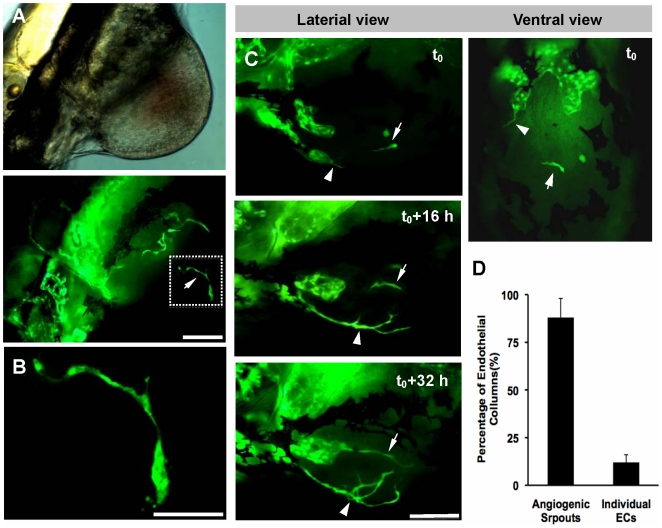
The participation of individual VEGFR2+ endothelial cells to the initial formation of tumor vascular network. Individual VEGFR2+ endothelial cells located at the center of B16 xenograft (indicated by arrow, A). The region in white dotted box was magnified in B. With the tumor growth (C), individual endothelial cells (arrows) increased its length and fused with angiogenic sprouts (arrowheads). (D) Histogram represents (n = 12, p<0.05) the percentage of endothelial columns that originated from the individual ECs or the angiogenic sprouts. Scale bar, 100 µm (A, C) or 50 µm (B).

Through careful investigating the formation of the initiation vascular network, we found there were two distinct neovascularization processes occurred in B16 xenogarfts (Fig. 2 and 3). First, in all B16 neoplasia (Fig. 2A), the initial neo-angiogenic sprouts firstly projecting from the preexisting host dilated vessels on 2nd day after cell injection (Fig. 2B, 2dpi panel). With the growth of B16 tumor (Fig. 2A), more neo-vessels were induced into tumor tissue and tumor vascular architectures were established gradually (Fig. 2A,B). On the 6th day post injection, the obvious tumor mass could observed in zebrafish (Fig. 2A, 6dpi panel), in which filled with a chaotic plexus of newly formed angiogenic sprouts (Fig. 2B, 6dpi panel). Second, in most B16 neoplasia (about 70%), some individual VEGFR2+ endothelial cells could be observed at the central region of B16 tumor xenografts (Fig. 3A,B). Dynamic real-time imaging revealed that the length of these individual VEGFR2+ endothelial cells increased gradually with the progression of tumor neovascularization and finally fused with adjacent growing neoangiogenic sprouts (Fig. 3C). Quantitative analysis indicated that angiogenic sprouts derived from host vessels had a major contribution to the initial formation of tumor vascular network, while those individual endothelial cells within microtumors have a relative less contribution to that (12±4%, n = 12) (Fig. 3D).

### The formation process of chaotic vascular network in B16 xenograft

**Figure 4:**
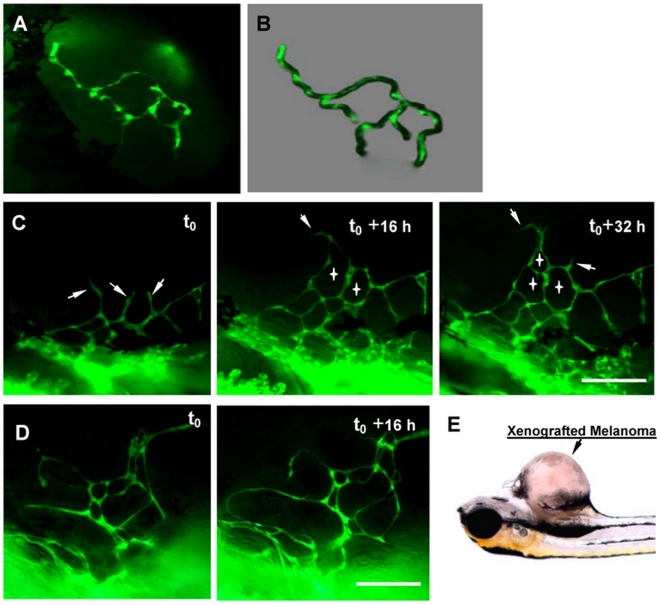
The formation of chaotic microvascular network in B16 xenografts. (**A**) The interconnection of initial endothelial cells that had penetrated into tumor tissue. (B) Schematic diagram showing the forming and formed endothelial loops. (C) The dynamic process of more and more endothelial loops assembling into a complex vascular network. Angiogenic sprouts were indicated by arrows, the formed endothelial loops were indicated by crosses. (D) Endothelial loops varied significantly in size and shape. The B16 xenograft is indicated by arrow (E). Scale bars, 200 µm.

Vascular network in malignant tumor uses to be highly irregular, heterogenic and tortuous. In order to illustrate how angiogenic sprouts grow into a complex and chaotic vascular network, the neovascularization in B16 melanoma xenografts in Tg(flk1:EGFP) transgenic zebrafish were dynamically imaged (Fig. 4 A–E). The observations showed that the initial endothelial cells that had penetrated into tumor tissue preferred to connect each other to form endothelial loops rather than continuously growing forward in tumor tissues (Fig. 4 A,B). This imaging possess unprecedented high-resolution that pseudopodia protruding from individual endothelial cells could be clearly displayed (Fig. 4 A). With the growth of the tumor, more and more endothelial loops assembled into a complex vascular network (Fig. 4C, angiogenic sprouts were indicated by arrows, the formed endothelial loops were indicated by crosses). In addition, endothelial loops were diverse significantly in size and shape (Fig. 4D). Consequently, the final vasculatures in tumors, comprising of diverse numerous endothelial loops, look like highly irregular and chaotic comparing to that in normal tissues.

### Significant effects of angiogenic gene VEGF165 to the neoangiogenesis in tumor xenografts

**Figure 5:**
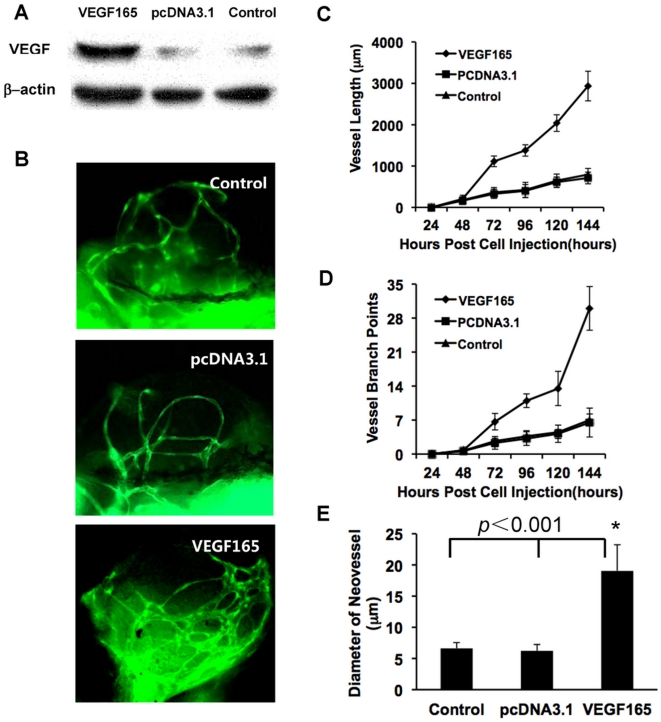
The over-expression of VEGF in B16 cells promoting tumor neovascularization. (A) Weston-blot revealed the expression of VEGF165 at 48 hours post transfection. (B) The tumor-induced neo-vessels were greatly affected by the over-expression of VEGF165 in B16 cells, (C–E) Statistical graphs revealed the changes of vessel length (C), vessel branch points (D) and mean diameters of neovessels (E). Scale bar, 100 µm.

To evaluate whether this xenograft model can be used for the investigation of angiogenic-relative genes, B16 cells over-expressing VEGF165 (Fig. 5A) were implanted into zebrafish. Non-transfected and vector transfected B16 cells were used as controls. On the 6th day post injection, in VEGF165 over-expressed B16 melanoma xenografts, in addition to more neovessels were induced into (Fig. 5B), vessel length, vessel branch points and vessel diameter also significantly increased based on the quantitative analysis, when compared to control B16 xenografts (Fig. 5B–E).

### Significant inhibition of tumoral neovascularization by SU5416

**Figure 6:**
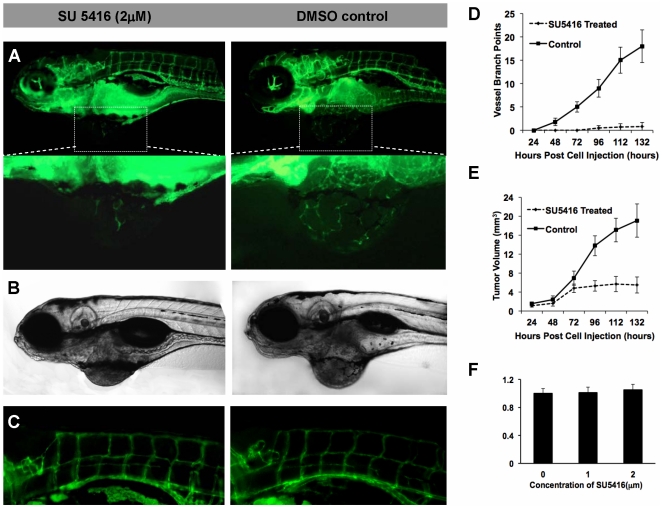
The small-molecular inhibitor of VEGFR2 inhibiting the tumor growth and neovascularization. (A–C) The imaging showed the obvious blockage of the specific inhibitor of VEGFR2, SU5416 (2 µM), to the neovascularization (A), tumor growth (B), and normal vascular vessels of zebrafish (C). (D,E) The quantitative analysis of the inhibiting effects of SU5416 to neovascularization (D) and tumor growth (E). (F) The result of MTT assay for B16 cells that treated by SU5416 for 48 hours *in vitro* at a concentration of 0, 1, 2 µM. Scale bar, 100 µm.

To address the application of this xenograft model for the screening of chemical drug candidates, we investigated the effects of SU5416, a widely used VEGFR2 specific inhibitor, on the angiogenesis in B16 melanoma xenografts in zebrafish. The results showed that 2 µM SU5416 significantly inhibited the neovascularization (Fig. 6A) and tumor growth (Fig. 6B) of B16 xenografts on 5 dpi, however, had little effects to the normal vascular vessels of zebrafish (Fig. 6C). The quantitative analysis further confirmed the inhibiting effects of SU5416 to neovascularization (Fig. 6D) and tumor growth (Fig. 6E). To exclude the toxicity of SU5416 (2 µM) to B16 cells, we tested the proliferation rate of B16 cells (treated with or without SU5416) *in vitro* and did not found obvious inhibition (Fig. 6F).

## Discussion

Anti-angiognesis represents a promising strategy for cancer therapy. Zebrafish has several unique features including develop rapidly *ex vivo*, transparent body during the first week, inexpensive to maintain, breed in large numbers, and can be maintained in small volumes of water [23]. These characteristics have made zebrafish become an attractive model for cancer research and angiogenesis assay.

Previous studies have shown that transplantation of human tumor cell lines into zebrafish embryos induces increased vasculature, providing a model to investigate tumor xenografts induced neovascularization [18], [19]. However, tumor xenografts in these models are embedded in the zebrafish tissues, resulting in the difficulties of real-time monitoring tumor growth and direct observations of neovascularization within tumor tissues on single-cell level. Moreover, hosts (zebrafish) must be sacrificed to quantitatively analyze the neovascularization in xenografts. In the present study, we have described a novel experimental procedure to establish a tumor xenograft model for angiogenesis assay in Tg(flk:EGFP) transgenic zebrafish. Comparing to known models in rodent, chicken embyos and zebrafish, this one has some unique characteristics: first, the protocol is much easier and simpler, only need the direct injection of tumor cell suspension into zebrafish and no other reagents are included, such as Matrigel [18], [19]. Second, tumor xenografts in zebrafish grow locally in perivitelline space and finally form an isolated hemispherical neoplsia protruding out of the zebrafish body. So, the shape of xenografts is regular and obvious during the whole growth process, the tumor growth can be readily measured for quantitative analysis without the interference from the host tissues. Third, the sharp visual contrast between green vascular endothelial cells and red tumor cells within isolated and transparent xenografts provides unprecedented clarity allowing the clear observation of neovascularization at single-cell level. Fourth, no surgical operations are needed on the animals during the whole experimental process, so it is very appropriate for dynamic observations of neovascularization during tumor growth. Fifth, there are no any neovascularization in the region where tumor cells were implanted throughout the experimental period in the absence of inducement from tumor cells. Thus, almost all newly formed ectopic microvessels induced by tumor xenografts can be unequivocally identified and quantified under fluorescent microscope with unprecedented clarity. Consequently, with this model we have clearly investigated the initiation of vascularization within tumor tissues, and recorded the participation of VEGFR2^+^ individual endothelial cells. Furthermore, we have quantified the contributions of classical angiogenesis and VEGFR2^+^ individual endothelial cells to the formation of tumor vascular network. Our dynamic observations showed that these VEGFR2+ individual endothelial cells were able to increase their length over time and then fuse with the sprouting tumor vasculatures. Their behaviors are similar to endothelial precursor cells (EPCs) that have been previously described on mammalian tumor model [24], [25]. To our knowledge, at least, this is the first time that clearly *in vivo* observed the dynamic process of EPC-like endothelial cells participating in the formation of tumor neovaculature at the initial stage of tumor growth.

The neovasculature in tumor is chaotic, of heterogeneous angio-architecture, has large caliber vessels and sluggish blood flow [26]. However, the formation process of this complex vascular network *in vivo* is still not clear. The tumor xenografts in the present study initiated from a very limited number of cells (as low as 50–100 cells per embryo), thus can mimic the earliest stages of tumor angiogenesis. Based on continuously dynamic observations with high-resolution, we found endothelial cells that had penetrated into tumor tissue preferred to connect each other to form endothelial loops rather than continuously growing forward straightly. These endothelial loops were varied significantly in size and shape. The final vasculatures in tumors comprise of diverse numerous endothelial loops, thus look like highly irregular and chaotic comparing to that in normal tissues. These *in vivo* observations about the formation process of tumor vasculatures provide a reasonable explanation on cellular level to why the architecture of neovasculatures in tumor always is chaotic and heterogeneous. Also, these results provided a good clue to the molecular mechanisms underlying this process that need to be further investigated in the future work.

The development of vascular system in zebrafish embryo shows strong similarity to that in other vertebrates [20], [21]. Robust angiogenic responses can be triggered in zebraifsh when human and murine tumorigenic cell lines were injected into 2-day-old zebrafish embryos [18]. Furthermore, the zebrafish tumor xenograft models have shown the capacity to discriminate between highly and poorly angiogenic tumor cells in previous [18] and our present studies, indicating zebrafish tumor xenograft models can be used to investigate the mechanisms of cancer neovascularization. Previous studies [18], [19] have shown that tumorigenic FGF2-overexpressing mouse aortic endothelial cells (FGF2-T-MAE) triggered the rapid formation of a new microvasculature when grafted close to the developing subintestinal vessels (SIV) of zebrafish embryos at 48 h postfertilization, whereas parental MAE cell grafts only limited change the SIV architecture. In the present study, we showed that VEGF-overexpressing B16 cells induced stronger angiogenic response (in vessel length, vessel branch points and vessel diameter) within tumor xenografts. Obviously, comparing to other often used angiogenesis assays, such as rodents cornea or a chick embryo chorioallantoic membrane [27]–[29], zebrafish xenograft model has unique characteristics: first, this is an *in vivo* model but assay can be very easily finished as an *in vitro* manner. Second, the usage of Tg(flk1: EGFP) make neo-vessels within tumor tissues can be easily observed and quantified. Therefore, through injecting distinct cells, in which target genes have been over-expressed or down-regulated into zebrafish embryos as the approach described in our present study, we can readily identify angiogenesis-relative genes underlying angiogenic process.

In contrast to the mouse, the zebrafish is a more economical and efficient model species for the cost-quality and high-throughput *in vivo* screening of small-molecule anti-tumor compounds, in that: 1) zebrafish embryo shows great permeability to small-molecule chemical compounds [30]. 2) The maintenance cost of zebrafish is less than 1% that of mice [31]. 3) A large number of tumor xenograft in zebrafish embryos can be easily established. A trained researcher is capable to inject tumor cells into 100–200 embryos within one hour. 4) Zebrafish with tumor xenograft can be maintained in six- or 96-well plates, so the systemic *in vivo* screening can be performed with minimal amounts of compound. Also, dose–response experiments of numerous compounds can be easily performed. Importantly, the zebrafish tumor model can investigated simultaneously the toxicity of tested compounds to normal vessels and embryo development. We used a well-known chemical compound SU5416, the specific inhibitor of VEGFR2, as the model drug to validate the application of this xenograft. 2 µM SU5416 significantly inhibited the angiogenesis within B16 xenografts on 5 dpi, but had little effects to the normal vascular vessels of zebrafish and the embryo development. The experiment was performed in a 96-well plate (100 µL per well), so only a small quantity of chemical inhibitor was used. The whole experimental process is similar to the tests on cultured cells *in vitro*, but that we obtain are *in vivo* data on the whole animal level.

Taken together, the zebrafish tumor xenograft model established in the present study represents a promising *in vivo* platform for tumor angiogenesis investigation and anti-tumor drug discovery.
